# Echinococcal disease in Alberta, Canada: more than a calcified opacity

**DOI:** 10.1186/1471-2334-5-34

**Published:** 2005-05-17

**Authors:** Ali Somily, Joan L Robinson, Lilly J Miedzinski, Ravi Bhargava, Thomas J Marrie

**Affiliations:** 1 Department of Pathology, University of Alberta, Edmonton AB, Canada; 2 Department of Pediatrics And Stollery Children's Hospital, University of Alberta, Edmonton AB, Canada; 3 Department of Medicine, University of Alberta, Edmonton AB, Canada; 4 Department of /Radiology, University of Alberta, Edmonton AB, Canada

## Abstract

**Background:**

Most cases of echinococcal disease (ED) acquired in Canada are thought to be due to the sylvatic form of *Echinococcus granulosus*, which may be more benign than ED due to either *Echinococcus multilocularis *or the pastoral form of *E. granulosus*. There are limited descriptions of the clinical course and outcome of Canadian patients with ED in the modern era.

**Methods:**

A retrospective chart review was performed of patients hospitalized with echinococcal disease (ED) from 1991 to 2001 in Edmonton, Alberta.

**Results:**

Forty-two cases of ED were identified of which 19 were definite, 3 probable, and 20 possible. Further analysis was limited to the 22 definite and probable cases, of which 77% were female and 41% aboriginal, with an age range of 5 to 87 years. Nine patients (40%) had pulmonary involvement and 11 (50%) hepatic involvement. One patient had an intracardiac mass presenting as a cerebrovascular event and one had a splenic cyst. Seven of the 22 patients had combined surgical resection and medical treatment, six had surgical resection of the cyst alone, four had cyst aspiration, one had medical treatment alone and four had no specific treatment. There was no mortality attributable to ED but three patients died of unrelated illnesses.

**Conclusion:**

Echinococcal disease in northern Alberta has a marked diversity of clinical presentations, and generally has a good prognosis despite a wide variety of therapeutic interventions.

## Background

Echinococcal disease (ED) is a zoonotic infection caused by cestodes of the genus *Echinococcus*. Alveolar echinococcosis results from infection with the species *Echinococcus multilocularis*. Disease occurs in the Northern Hemisphere including Alaska, Japan, China, Russia and most countries in continental Europe [1], and results in multilocular cystic lesions. Cystic echinococcosis (CE) results from infection with the species *Echinococcus granulosus*. Disease occurs worldwide with the highest incidence in Mediterranean countries, Russia and the adjacent newly independent states, China, north and east Africa, Australia, and South America [1]. *Echinococcus granulosus *infections have been further classified as pastoral or sylvatic variants or biotypes. In the pastoral variant, sheep serve as the most common intermediate hosts while in the sylvatic variant the usual intermediate hosts are caribou or moose.

Most cases of ED acquired in Canada are thought to be due to the sylvatic form of *E. granulosus*. In the Northwest Territories and the northern portion of the Canadian Prairie Provinces of Alberta, Saskatchewan and Manitoba, the barren ground caribou is the most common intermediate host [2]. Moose with hydatid cysts have been found in every province of Canada west of the Maritimes and it is estimated that 50% of moose in Ontario and British Columbia are infected with the parasite [3]. Hydatid cysts have also been found in wapiti, elk, reindeer, coastal deer, white tail deer and bison. The definitive host is a canine (dog, fox, or wolf) that harbors the tapeworm after ingestion of tissue from an infected intermediate host. Twenty-eight to fifty percent of dogs in the Northwest Territories are infected with *E. granulosus *[2]. Intermediate hosts or humans can ingest eggs shed in the feces of the definitive host, with humans being dead-end hosts. Ingestion of *E. granulosus *eggs by intermediate hosts or humans is followed by the release of an oncosphere; the larva is subsequently transported via the blood stream or lymphatics to primary target organs (the lung or liver, and rarely the central nervous system, bone, or myocardium). Within the affected organ, the oncosphere matures into a vesicle, which grows expansively by concentric enlargement. A fully mature hydatid cyst, normally fluid-filled and unilocular, represents the final stage [4].

Edmonton is a referral center for northern Alberta and the Northwest Territories, thereby affording us an opportunity to see the complications of hydatid disease. We reviewed a decade of our experience with ED to gain further understanding of the epidemiological and clinical features of this disease.

## Methods

A retrospective chart review was performed of all cases identified as ED using International Classification of Disease (ICD) Ninth Edition codes [ICD-9 122.0 to 122.9] from 1991 – 2001 in all Edmonton regional hospitals. Demographic, clinical and diagnostic data were collected. Aboriginal persons were defined as self-identified native Indian or Inuit people, or individuals with addresses that were on reserve land.

Definite ED was defined by evidence of ED on pathology (hooklets, brood capsules, or parasitic degenerative elements), probable ED by positive serology for *Echinococcus *and possible ED by a clinical presentation compatible with ED but with no corroborative histologic or serologic evidence.

Ultrasounds of all cystic lesions from patients who met the study criteria were reviewed by a radiologist who was blinded as to whether the patients had possible, probable, or definite ED. Cysts were then classified according to the proposed international classification system [5].

## Results

### Illustrative case

A 28-year-old aboriginal female who was five days postpartum had productive cough, pleuritic chest pain, dyspnea, fever, chills and sweating, and had received cephalexin, ciprofloxacin and cloxacillin without clinical improvement. She lived in an aboriginal community and had a history of contact with dogs and the ingestion of meat from a variety of wild animals. Laboratory results included a white blood cell count of 13.3 × 10^9^/L, with 1.8 × 10^9^/L (14%) eosinophils, erythrocyte sedimentation rate of 47 mm/hr, AST 32 U/L, and ALT 112 U/L. The chest radiograph showed a cavity in the left lower lobe and lingula with an air fluid level. Computed tomography of the chest showed a solitary (4.5 × 6.3 × 7.7 cm) lingual cavity with calcification, septation and a small amount of fluid with an opacity in the posterior segment of apico-posterior segment of the left upper lobe in addition to a left pleural effusion.

The patient underwent thoracotomy and left segmental pneumonectomy, left lingulectomy and decortication of the lung. Histologic examination of the lung cysts showed occasional hooklets and degenerative parasitic elements. She had a relatively uncomplicated post operative recovery and was discharged on albendazole 400 mg PO bid for 4 weeks. Echinococcal serology revealed an antibody titer to *Echinococcus *of 1:1024 using an Enzyme-linked immunosorbent assay (ELISA) technique.

## Review of cases

### Demographics

Forty-two cases of ED were identified, of which 19 were classified as definite, 3 as probable, and 20 as possible. The demographic features are shown in Table 1. Of the 22 definite and probable cases, 77% were female and 41% were aboriginal. The mean age of the definite and probable cases was 32 years (range 5 – 87 years). Possible cases were predominantly male (55%) and older (mean age 54 years). Eight of the total 42 patients had a history of eating caribou, elk or moose meat. Seven patients had a history of contact with dogs, and none had a history of contact with foxes or wolves. Unfortunately this information was not consistently recorded in the patient records.

**Table 1:** Demographic features of 42 patients hospitalized in Edmonton, Alberta with echinococcal disease 1991–2001

	Definite/Probable (N = 22)	Possible (N = 20)	Total (N = 42)
Male	5 (23%)	11 (55%)	16 (38%)
Female	17 (77%)	9 (45%)	26 (62%)
Born in Canada	14 (64%)	14 (70%)	28 (67%)
Foreign born*	4 (18%)	2 (10%)	6 (14%)
Place of birth unknown	4 (18%)	4 (20%)	8 (19%)
Edmonton residence	6 (27%)	6 (30%)	12 (29%)
NWT residence	9 (41%)	8 (40%)	17 (40%)
Rural Alberta residence	7 (32%)	6 (30%)	13 (31%)
Mean age (years)	32 ± 16	54 ± 25	
Pulmonary disease	9 (40%)	2 (10%)	11(26%)
Hepatic disease	12 (55 %)	16 (80%)	28 (67%)
Spleen /kidney	0	2 (10%)	2 (5%)
Cardiac	1 (5%)	0	1 (2%)

*Patients were born in Lebanon, Ukraine, Iraq, Paraguay, Poland, or Yugoslavia

Of the 22 definite and probable cases of ED, 9 (40%) had pulmonary involvement, 11 (50%) had hepatic involvement, one (5%) had splenic involvement and one (5%) had cerebral disease. The patient with cerebral disease was a 28-year-old woman with cerebral emboli, who had a pre-operative diagnosis of cardiac myxoma based on echocardiography and magnetic resonance imaging. The cardiac mass was resected and found to be an echinococcal lesion. Ten percent of the possible ED patients had pulmonary lesions, 80% had hepatic lesions, and 10% had non-hepatic intra-abdominal lesions (an immigrant from Poland with a calcified 10 cm diameter cyst in the spleen, and a man with adenocarcinoma of the rectum who had several calcified cysts in the right kidney).

The presenting symptoms of the 38 patients with pulmonary or hepatic disease are shown in Table 2. Predominant symptoms were pleuritic chest pain (82%), cough (73%), and dyspnea (56%) in the patients with pulmonary involvement, and abdominal pain (64%) in the patients with hepatic involvement.

**Table 2:** Symptoms in 38 patients with echinococcal disease and pulmonary or hepatic involvement

	**Pulmonary**		**Hepatic**	
**Symptom**	Definite/probable (N = 9)	Possible (N = 2)	Definite/probable (N = 11)	Possible (N = 16)

Asymptomatic	0	0	2	2
Weight loss	2	2	1	2
Pleuritic chest pain	7	2	1	2
Cough	6	2	1	4
Hemoptysis	1	1	0	1
Dyspnea	5	1	2	4
Abdominal pain	0	0	9	9
Fever	3	0	4	5
Night sweats	2	0	1	2
Anorexia	4	0	1	4
Malaise	2	1	1	2
Jaundice	2	0	1	2
Chills	1	0	3	1
Vomiting	4	0	2	4

### Investigations

Results of laboratory investigations in the 38 patients with pulmonary or hepatic disease are shown in Table 3. Mild anemia was common, and 37% of the 19 patients with definite and probable ED who had a differential white blood cell count performed had eosinophilia. Of the12 patients with definite and probable pulmonary or hepatic ED who had ELISA testing performed, six had positive results. Testing was performed using a titer method (four of seven were positive) or an optical density method (two of five were positive).

**Table 3:** Mean values and ranges of results of laboratory investigations in 38 patients with pulmonary or hepatic echinococcal disease *

	**Pulmonary**		**Hepatic**	
**Laboratory test** **Normal values**	Definite/probable (N = 9)	Possible (N = 2)	Definite/probable (N = 11)	Possible (N = 16)

Hemoglobin 135 – 175 g/L	120(112–133)	114.5(113–116)	123.7 (96–180)	124 (93–161)
White blood cell count 4–11 × 10^9 ^/L	10(6.4–18)	9.7(9.6–9.8)	8.7 (4.3–14.5)	8.26 (3.8 – 14)
Eosinophilia >= 0.1 × 10^3 ^/L	2(0.1–7)	1.95(0.3–3.6)	1.8 (0 .1–6)	1.5 (0 .1 – 4.7)
ESR 0–20 mm/hr	28.5(10 – 47)	30.5(28–33)	32.6 (18–47)	23 (11–35)
AST <40 U/L	30.4 (14–49)	25.5(22–29)	32 (12–59)	63 (14 – 432)
Alkaline Phosphatase 30–130 u/L	122.3(48 – 231)	137(123–152)	141 (62 – 461)	168 (41 – 892)
Total Bilirubin 3.4–17.1 umol/l	11(4–27)	4.5(2–7)	15.7 (4–32)	24.4 (3.3 – 98)
ELISA (positives/ number tested)	3/7	0/2	3/5	0/5

Legend: ELISA – enzyme-linked immunosorbent assay; ESR – erythrocyte sedimentation rate

* Results are incomplete in some patients.

Results of chest radiographs for the 38 patients with pulmonary or hepatic involvement are shown in Table 4. Six of the 11 lesions in the patients with pulmonary involvement were in the left lung with either a cystic mass (present in nine of 11 cases) or nodular appearance associated with a pleural effusion in five of nine pulmonary cases. One patient had a pneumonic presentation with no cyst. Four of the 27 patients with hepatic disease had multiple liver cysts. The right lobe of the liver was involved in about two-thirds of the hepatic cases.

**Table 4:** Chest radiographic findings in 42 patients with echinococcal disease

	**Pulmonary**		**Hepatic**		**Other**	
**Chest radiograph**	Definite/probable N = 9	Possible N = 2	Definite/ probable N = 11	Possible N = 16	Cardiac (definite) N = 1	Kidney or spleen (probable/possible) N = 3

Normal	0	0	5	7	0	1
Abnormal	9	2	6	9	1	2
Cyst	7	2	0	2	0	0
Pleural effusion	4	1	4	3	1	0
Pneumonia	5	1	0	4	0	0
Cavity	2	1	1	0	0	0
Granuloma	0	0	1	0	0	0
Atelectasis	1	0	1	3	0	1
Calcified cyst	6	2	0	1	0	0

Ultrasounds were available for review in 15 of the 30 patients with intra-abdominal disease. Results are shown in Table 5 with seven showing active cysts (CE1 or CE2) and eight showing inactive cysts (CE 4 or CE5) [5].

**Table 5:** Ultrasound findings in 15 patients with intra-abdominal cystic echinococcosis graded according to the proposed international classification (5)*

	Definite/probable hepatic (N = 4)	Possible hepatic (n = 8)	Possible kidney (N = 1)	Probable splenic (N = 1)	Possible splenic (N = 1)
CL	-	-	-	-	-
CE1	-	3	1	-	-
CE2	1	2	-	-	-
CE3	-	-	-	-	-
CE4	3	-	-	1	1
CE5	-	3	-	-	-

*Ultrasounds were no longer available for review on the other 15 patients with intra-abdominal disease.

### Therapy and outcome

Treatment of all patients with is shown in Table 6. Of the 22 patients with definite and probable ED, seven had surgical resections of cyst(s) combined with medical treatment (albendazole or mebendazole), six had surgical removal of the cyst alone, four had cyst aspiration, one had medical treatment alone and four were not specifically treated. Of the 20 cases with probable ED, one had surgical resections of cyst combined with medical treatment, one had surgical removal of the cyst alone, one had medical treatment alone and 17 were not specifically treated. Pre-operative complications included suspected rupture of a cyst into a bronchus in two patients, suspected bacterial infection of the cyst in two patients with pulmonary cysts and one patient with a hepatic cyst, and obstructive jaundice in one patient. Major complications after surgical resection occurred only with hepatic disease and included single cases of pleural effusion, wound infection, biliary leak, and intra-abdominal bleeding resulting in a hepatic lobectomy.

**Table 6:** Therapy of patients with hydatid disease

	**Pulmonary**		**Hepatic**		**Other**	
**Therapy**	Definite/probable N = 9	Possible N = 2	Definite/probable N = 11	Possible N= 16	Cardiac (definite) N = 1	Kidney or spleen (probable/possible) N = 3

Medical only*	0	1	1	0	0	0
Surgical resection only	3	0	2	1	1**	0
Medical therapy and surgical resection	5	0	2	1	0	0
Aspiration of cyst only	0	0	4	0	0	0
Aspiration of cyst and medical treatment	0	0	0	0	0	0
Observation only	1	1	2	14	0	3

* mebendazole or albendazole

**echinococcal cyst was resected from left ventricle

Three patients with possible hepatic ED died of illnesses that were not directly related to ED. The first patient had cryptogenic cirrhosis and chronic cholelithiasis, as well as a left lobe liver cyst. She had an open cholecystectomy for acute cholecystitis, and died post-operatively from intra-abdominal bleeding and sepsis (pathology was not performed on the hepatic cyst). The second patient with multiple myeloma and paraplegia was incidentally noted to have a hepatic cyst and died of unexplained respiratory failure. The third patient admitted with bilateral deep vein thromboses and incidentally found to have hepatic cysts died of bronchopneumonia and heart failure.

### Comparison of Canadian-born to foreign-born patients

In comparing the clinical features and outcome of Canadian-born patients (presumed to have the sylvatic variant of *E. granulosus*) and foreign-born patients (who could have any type of *Echinococcus *infection), no differences were noted (data not shown).

## Discussion

Over a ten-year period there were 19 definite, 3 probable, and 20 possible cases of ED identified at a northern Alberta referral center. In North America, ED was previously considered a disease of immigrants. Of 596 cases of ED reported up to 1950, only 36 occurred in persons born in Canada or the United States [3]. In our series, only 6 of 36 cases where the country of birth was known were foreign-born. Forty-one percent of the 42 cases occurred in aboriginal Canadians, who represent only 5.3% of the population of Alberta and 3.4% of the population of Canada (2001 Canadian census data). The majority of patients were middle-aged females. Previous studies have shown a predominance of males [4] and females [6], and it has been postulated that the sex predominance may be determined by which sex has more contact with the usual definitive host in that country [3].

Two-thirds of the cases of ED in our study were pulmonary, but for the definite and probable cases, half were hepatic. A previous study from northern Canada and the United States described hepatic involvement in 71% of cases and pulmonary involvement in only 7% of cases [4]. The results of a recent study of 17 patients from Manitoba and northwestern Ontario were comparable to ours with pulmonary involvement in 47% and hepatic involvement in 47% of cases [7]. However, the true rate of hepatic versus pulmonary ED cannot be determined by these studies as detection is often on the basis of radiographic studies performed for reasons unrelated to the ED. Previous reviews suggested that pulmonary cysts are more common in children and young adults while hepatic cysts are more common in older persons [2], which is compatible with our findings (data not shown).

Laboratory tests are of limited value in the diagnosis of ED. One study showed that only 29% of cases with pulmonary cysts and 15% of cases with hepatic cysts had eosinophilia [8]. In our series, 54 % of the pulmonary cases and 22% of the hepatic cases had eosinophilia. Perhaps this low rate is explained by the chronicity of the disease with eosinophilia being present only early in the course via stimulation of Th2 lymphocytes and production of IL-4 and IL-5 subsequently generating IgG1 and IgE-secreting cells, and eliciting eosinophilia [9,10]. Another possibility is that eosinophilia is only likely after cyst rupture and release of antigenic material [11].

Serology for ED has variable sensitivity depending on the type of test. The most sensitive is IgG ELISA with an 83% sensitivity [10] although this test may only become positive after cyst rupture [11]. In our study, serology was positive in 54% of cases of definite and probable ED.

There are no clinical features that can reliably distinguish *E. granulosus *from *E. multilocularis*. However, a specific *E. multilocularis *antigen such as the high affinity purified Em2 antigen AE metacestodes has been reported to be able to discriminate between *E. granulosus *and *E. multilocularis *in 95% of cases [12]. This test was not available at the time of this review. Molecular methods to distinguish the pastoral from the sylvatic form of *E. granulosus *have recently been described [13]. Daughter cysts have not been reported in sylvatic disease [14], and this form of the disease appears to be more benign than the pastoral form with a far lower incidence of complications even when cyst rupture occurs [14]. Most cases of the sylvatic form of ED are asymptomatic and are diagnosed when cysts are found during procedures performed for other reasons [14]. It seems likely that most of our Canadian-born patients had sylvatic disease as they reside in areas where exposure to sheep is rare. Most of our patients with pulmonary cysts were minimally symptomatic on presentation with cough or chest pain, and cyst rupture was recognized in only two pulmonary cases. In addition, four patients with definite and probable ED were discharged with no therapy and had no apparent sequelae.

Traditionally, surgical resection of the cyst was the treatment for ED, but simpler surgical procedures such as hydatid cyst aspiration have been shown to result in low mortality and improved outcome [6]. However, medical management of relatively asymptomatic presumed sylvatic cases can be considered, as the natural history of the sylvatic variant is to rupture without any complications or anaphylaxis [14]. It is not clear if albendazole or mebendazole are indicated or if observation is sufficient. Surgical resection or cyst aspiration is recommended in sylvatic cases only for complications such as secondary infection or pressure symptoms.

Although the outcome of our patients was generally good complications did occur. Two patients had symptomatic rupture of a pulmonary cyst and two patients had suspected bacterial infection of pulmonary cysts. Pressure or mass effects of hepatic cysts on the bile ducts, portal and hepatic veins, or on the inferior vena cava can result in cholestasis, portal hypertension, venous obstruction, or Budd-Chiari syndrome, sometimes resulting in the need for liver transplantation [15]. Obstructive jaundice was described in 18% of 132 patients in one case series [16] and occurred in one of our 12 definite and probable hepatic cases. Rupture of cysts into the biliary tree can produce biliary colic, obstructive jaundice, cholangitis or pancreatitis [17]. Cerebral disease is more common in children than in adults, and occurs in 1.5%–2.0% of patients, with the most common presentations being seizures or signs of raised intracranial pressure secondary to the cysts in the brain [7]. In our single case, cerebral disease was due to emboli from a cardiac echinococcal lesion.

## Conclusion

Prevention of ED has been attempted by distributing praziquantil bait in areas where there is a high prevalence of infected foxes [18]. Unfortunately, such strategies are not practical in a vast country such as Canada. Until our understanding of the modes of transmission of ED improves, education of the public about this relatively rare disease is unlikely to be effective. Therefore, from a public health perspective, the emphasis must be on further progress in management of this disease, which will require increasing awareness on the part of physicians regarding the multitude of presentations, improved diagnostics, and further study of long-term outcomes with different treatment modalities.

## Competing interests

The author(s) declare that they have no competing interests.

## Authors' contributions

All authors contributed to study design and to revising the manuscipt. AS collected and analyzed the data and wrote the manuscript.

## Pre-publication history

The pre-publication history for this paper can be accessed here:


